# Obesity-Induced Dysbiosis Exacerbates IFN-γ Production and Pulmonary Inflammation in the *Mycobacterium tuberculosis* Infection

**DOI:** 10.3390/cells10071732

**Published:** 2021-07-08

**Authors:** Sandra Patricia Palma Albornoz, Thais Fernanda de Campos Fraga-Silva, Ana Flávia Gembre, Rômulo Silva de Oliveira, Fernanda Mesquita de Souza, Tamara Silva Rodrigues, Isis do Carmo Kettelhut, Camila Sanches Manca, Alceu Afonso Jordao, Leandra Naira Zambelli Ramalho, Paulo Eduardo Martins Ribolla, Daniela Carlos, Vânia Luiza Deperon Bonato

**Affiliations:** 1 Basic and Applied Immunology Program, Ribeirao Preto Medical School, University of Sao Paulo, Ribeirao Preto, Sao Paulo 14049-900, Brazil; sandrapalma3@gmail.com (S.P.P.A.); thaisfragasilva@gmail.com (T.F.d.C.F.-S.); deoliveira.romulo@gmail.com (R.S.d.O.); fernandams@usp.br (F.M.d.S.); tamararodrigues@usp.br (T.S.R.); danicar@usp.br (D.C.); 2 Department of Biochemistry and Immunology, Ribeirao Preto Medical School, University of Sao Paulo, Ribeirao Preto, Sao Paulo 14049-900, Brazil; flagembre@yahoo.com.br (A.F.G.); idckette@fmrp.usp.br (I.d.C.K.); 3 Department of Internal Medicine, Ribeirao Preto Medical School, University of Sao Paulo, Ribeirao Preto, Sao Paulo 14049-900, Brazil; camila.nutriunifal@gmail.com; 4 Department of Health Sciences, Ribeirao Preto Medical School, University of Sao Paulo, Ribeirao Preto, Sao Paulo 14049-900, Brazil; alceu@fmrp.usp.br; 5 Department of Pathology and Legal Medicine, Ribeirao Preto Medical School, University of Sao Paulo, Ribeirao Preto, Sao Paulo 14049-900, Brazil; lramalho@fmrp.usp.br; 6 Biotechnology Institute, Sao Paulo State University, Botucatu, Sao Paulo 18607-440, Brazil; p.ribolla@unesp.br

**Keywords:** gut–lung axis, microbiota, obesity, tuberculosis

## Abstract

The microbiota of the gut–lung axis affects local and far-reaching immune responses and might also trigger chronic and inflammatory diseases. We hypothesized that gut dysbiosis induced by obesity, which coexists in countries with a high tuberculosis burden, aggravates the host susceptibility and the pulmonary damage tolerance. To assess our hypothesis, we used a model of high-fat diet (HFD)-induced obesity, followed by infection of C57BL/6 mice with *Mycobacterium tuberculosis*. We showed that obesity increased the susceptibility, the pulmonary inflammation and IFN-γ levels in *M. tuberculosis*-infected mice. During the comorbidity obesity and tuberculosis, there is an increase of Bacteroidetes and Firmicutes in the lungs, and an increase of Firmicutes and butyrate in the feces. Depletion of gut microbiota by antibiotic treatment in the obese infected mice reduced the frequencies of CD4^+^IFN-γ^+^IL-17^−^ cells and IFN-γ levels in the lungs, associated with an increase of *Lactobacillus*. Our findings reinforce the role of the gut–lung axis in chronic infections and suggest that the gut microbiota modulation may be a potential host-directed therapy as an adjuvant to treat TB in the context of IFN-γ-mediated immunopathology.

## 1. Introduction

The hallmarks of the gut–lung axis (GLA) are complex interactions from gut and lung microbiota combined with local and far-reaching immune effects [1,2]. Although gut microbiota has been extensively investigated, the detection of microbial DNA in the lungs of individuals in steady-state condition and the description of bacterial communities shared between the lung and the oral cavity were also reported [3]. The gut and respiratory tract show substantial differences, mainly in their functions and microenvironment. However, they share anatomical similarities, as both derive from the endoderm, present columnar epithelial cells with projections of microvilli (gut) or cilia (lung) and secrete mucus as well as immunoglobulin A [1].

Infectious or non-infectious chronic diseases might modify the interaction of the GLA. Invading microorganisms modify the microbiota and disrupt immune homeostasis, causing inflammatory disturbance within and outside the gastrointestinal tract [4]. The modification in composition and function of microbiota is described as dysbiosis, and may result in dislocation via circulation of microbial components and metabolites derived from the gut to the lungs, and modulate immune response [5,6,7,8].

Tuberculosis (TB) affects more than 10 million individuals and causes a major number of deaths worldwide [9], as a consequence of infection caused by a single infectious agent, *Mycobacterium tuberculosis*. It was reported that the infection with *M. tuberculosis* changed the composition of microbiota in a very rapid way (6 days post-infection) when comparing pre-infected BALB/c mice to infected surviving mice [10]. Interestingly, the antibiotic cocktail used to treat TB induced gut dysbiosis [11,12]. On the contrary, alteration in the gut microbiota driven by antibiotics increased the susceptibility to *M. tuberculosis* infection compared to mice with intact microbiota [13,14]. Therefore, either *M. tuberculosis* may affect gut microbiota or gut dysbiosis may induce progression of TB. 

Obesity is a global public health problem, which is closely associated with dysbiosis and type 2 diabetes mellitus (DM2) [15]. The development of TB is three times higher in subjects with DM2 that exhibit a worse outcome compared to those one with TB only [16]. Besides, obesity induces meta-inflammation, a low-grade and chronic inflammation [17]. Subjects with DM2 and TB exhibit elevated frequencies of Th1 and Th17 cells and decreased regulatory (Treg) cells compared with non-DB2 TB subjects [16,18]. Although IFN-γ is the golden standard of protection against tuberculosis [19,20], increased levels of IFN-γ induce type 1 inflammation and immunopathology, deleterious and associated with severe tuberculosis, as previously reported in clinical and experimental tuberculosis studies [21]. In the present study, we used a high-fat diet-induced obesity model to investigate the role of gut dysbiosis in the pulmonary inflammation in an attempt to elucidate key events that connect immunopathology and progression of infection. We hypothesized that obesity-induced gut dysbiosis exacerbates IFN-γ-mediated immunopathology, which is detrimental for host protection. 

## 2. Materials and Methods

### 2.1. Mice

Specific pathogen-free female C57BL/6 mice, 6–8 weeks old, were obtained from the breeding facility and were maintained in a biosafety level 3 facility in microisolator cages. Animals received a low- (LFD) or high-fat diet (HFD) and autoclaved water (for 12 weeks). Mice were weighed weekly. All animal procedures were carried out in accordance with the local Ethics Committee (protocol number 141/2014).

### 2.2. LFD and HFD

The HFD was modified from AIN-93G and consisted of 35% of lipids, 4% of vegetable origin (soybean oil) and 31% of animal origin (healthy pork fat). The total amount of calories in the HFD contained 15.1% kcal protein, 26.1% kcal carbohydrate and 58.7% kcal lipid. The control groups received a normolipid diet according to AIN-93G (LFD), which contained 20.5% kcal of protein, 63.6% kcal carbohydrate and 15.9% kcal lipid. 

### 2.3. Glycemia

After eight weeks of the LFD or HFD condition, the glucose levels were measured in the blood, obtained from the lateral tail vein after six hours of fasting, using a commercial glucometer (Accu-Chek Performa Nano, Roche Diabetes Care, Indianapolis, IN, USA).

### 2.4. Adipose Tissue Weight

After eight weeks of the LFD or HFD condition, the animals were euthanized with an overdose of ketamine-xylazine and the mesenteric adipose tissues were collected from lean and obese mice and weighed.

### 2.5. M. tuberculosis Infection

H37Rv *M. tuberculosis* strain (American Type Culture Collection 27294, Rockville, MD, USA) was grown in Middlebrook 7H9 broth (Becton Dickinson and Company, Sparks, MD, USA) for 10 days at 37 °C, and bacterial load was adjusted using McFarland standard. Mice were anaesthetized by intra-peritoneal injection of 100 μL of sterile saline solution containing 20% ketamine and 10% xylazine. Next, mice were infected by administration of 1 × 10^5^ bacilli by the intra-tracheal route, as previously reported [22], and evaluated at 30 days post-infection. 

### 2.6. Colony-Forming Unit Assay

For the colony-forming unit (CFU) assay, serial dilutions (102–105) of digested lower and middle right lobes of the lungs were plated on 7H11 agar. This agar was prepared using 0.47% Middlebrook 7H9 broth (Becton Dickinson and Company, Sparks, MD, USA) supplemented with 1.7% agar, 0.2% glycerol and 10% fetal bovine serum (Gibco, Invitrogen, Waltham, MA, USA). The CFU number was counted after 28 days of incubation at 37 °C [23].

### 2.7. Histopathology

The upper right lobes of the lungs were fixed in 10% formalin, embedded in paraffin blocks and cut into 5 µm thick sections. Lungs specimens were stained with hematoxylin and eosin (H&E). Pulmonary inflammation score was determined as: degree 0—absence or presence of rare inflammatory cells (without inflammation), degree 1—perivascular or peribronchial accumulation of inflammatory cells, predominantly lymphocytes, sometimes forming lymphoid aggregates (mild inflammation), degree 2—perivascular or peribronchial accumulation of inflammatory cells, predominantly lymphocytes and xanthomatous macrophages forming frequently lymphoid aggregates, sometimes coalescing and preserving alveolar spaces (moderate inflammation), and degree 3—perivascular or peribronchial accumulation of inflammatory cells, predominantly lymphocytes and xanthomatous macrophages forming frequently lymphoid aggregates, mostly coalescing and preserving rare alveolar spaces (severe inflammation).

### 2.8. Gene Expression

Total RNA was extracted from the lung and ileum tissues using Trizol (Invitrogen, Carlsbad, CA, USA). cDNA synthesis was performed using Reverse Transcriptase SuperScript II (Invitrogen, Carlsbad, CA, USA). Real-time PCR was performed in the StepOnePlus Real-Time PCR System (Applied Biosystems, Foster City, CA, USA), as previously described. Gene expression was calculated as 2^−ΔΔCT^, where ΔΔCt = ΔCt (sample) − ΔCt (calibrator), and where ΔCt (sample) = Ct (target gene) − Ct (normalizer = 2β microbulin). The following primer sequences were used for MMP-1, forward: GCC CAG AGA AAA GCT TCA GCA and reverse: TAG CAG CCC AGA GAA GCA ACA; *claudin-2*, forward: GGCTGTTAGGCACATCCAT, reverse: TGGCACCAACATAGGAACTC; 2β-microbulin, forward: CACCCCCACTGAGACTGATACTACATA and reverse: TCACATGTCTCGATCCCAGTAGA.

### 2.9. Cytokine Production 

TNF-α, IL-12, IL-1β, IFN-γ, IL-6, IL-17, IL-23 and TGF-β levels were determined at the left lobes of lung homogenates using the immunoassay kit BD OptEIATM set (BD Biosciences, San Jose, CA, USA), according to the manufacturer’s protocols.

### 2.10. Flow Cytometry

Lung cell suspensions (1 × 10^6^) from lower and middle right lobes were incubated for 40 min at 4 °C with the supernatant of 2.4G2 cell lineage (containing anti-FcγRII/II antibodies), followed by incubation for 30 min at 4 °C with monoclonal antibodies (mAb). For cytokine evaluation, lung cell suspensions were stimulated with PMA (100 ng/mL, Sigma-Aldrich, St. Louis, MO, USA), ionomycin (500 ng/mL, Sigma-Aldrich, St. Louis, MO, USA) and monensin (BD Biosciences, San Jose, CA, USA) for 4 h. The following mAb were used: anti-CD11b (M1/70), anti-Ly6G (IA8), anti-Ly6C (AL-21), anti-CD11c (HL3), anti-F4/80 (BM8), anti-CD16/32 (93), anti-MHCII (I-Ab 9/25/17), anti-CD103 (M290), anti-CD4 (RM4), anti-CD8 (53–67), anti-Foxp3 (MF 23), anti-IFN-γ (XMG1.2) and anti-IL-17 (TC11-18M 10.1), from Biolegend (San Diego, CA, USA). The samples were acquired using a BD FACSCanto II cytometer (BD Biosciences, San Jose, CA) and CellQuest software (Becton Dickinson and Company, Franklin Lakes, NJ, USA). One hundred thousand events per sample were collected and analyzed according to size, granularity and fluorescence intensity using FlowJo software (Becton Dickinson and Company, Franklin Lakes, NJ, USA).

### 2.11. Bacterial DNA Analysis

Bacterial DNA was isolated from fecal pellets using Kit QIAamp DNA Stool Mini (Qiagen, Hilden, NRW, Germany) and from lung samples using DNeasy Blood and Tissue Kits (Qiagen, Hilden, NRW, Germany) according to the manufacturer’s protocol. Quantitative PCR was performed and analyzed in the StepOnePlus Real-Time PCR System (Applied Biosystems, Foster City, CA, USA), as previously described. Differences (ΔCT) between cycle threshold (CT) values of bacterial 16S rRNA and (CT) values of specific bacterial groups were used to obtain normalized levels of each group (2^−ΔCT^). Relative abundance was obtained after normalization with the control group. The following primer sequences were used for detection: bacterial 16S, forward: AACAGGATTAGATACCCTGGTAG, reverse: GGTTCTTCGCGTTGCATC; Bacteroidetes, forward: GTTTAATTCGATGATACGCGAG, reverse: TTAASCCGACACCTCACGG; Firmicutes, forward: ATGTGGTTTAATTCGAAGCA, reverse: AGCTGACGACAACCATGCAC.

### 2.12. Short-Chain Fatty Acids’ Quantification

Short-chain fatty acids (SCFAs) were quantified in feces (150 mg) using a gas chromatographic (GC-2014, SHIMADZU, Columbia, MD, USA) method, that involves the extraction of the SCFAs in Milli-Q water before a direct injection procedure on a flame ionization detector using a silica capillary column (6890N GC, Agilent, Folsom, CA, USA). The procedures were adapted from Zhao [24].

### 2.13. In Vivo Intestinal Permeability Assay

FITC-labeled dextran (Sigma-Aldrich, St. Louis, MO, USA) was administered by oral gavage with a needle attached (440 mg/Kg body weight), as previously described [25]. Serum was collected retro-orbitally 4 h later and fluorescence intensity was determined by using the FlexStation^®®^ 3 Multi-Mode Microplate Reader (Molecular Devices, San Jose, CA, USA) (excitation, 485 nm; emission, 528 nm). Endotoxin was detected in serum after in vitro reaction with Limulus amebocyte lysate (LAL, QCL-1000TM Assay, Lonza, Morristown, NJ, USA), according to the manufacturer’s protocols.

### 2.14. Antibiotic Treatment and Fecal Transplantation

Mice received daily doses of a combination of 1.86 mg ampicillin (Sigma-Aldrich, St. Louis, MO, USA), 0.96 mg vancomycin (Sigma-Aldrich, St. Louis, MO, USA), 1.86 mg neomycin sulfate (Sigma-Aldrich, St. Louis, MO, USA) and 1.86 mg metronidazole (Sigma-Aldrich, St. Louis, MO, USA), diluted in 300 µL of drinking water by gavage for 21 days before *M. tuberculosis* infection in the HFD group. For LFD fecal transplantation, lean and obese mice were treated with antibiotics for 21 days, 6 days before infection and 15 days after infection. Mice treated with antibiotics were transplanted or not with fecal suspension to reconstitute the gut microbiota. Frozen fecal samples were collected from non-infected LFD and HFD groups, homogenized, suspended in water and supernatants were collected after centrifugation of the samples at 350 g for 2 min. Fecal transplantation was performed as follows: 5 doses in a 3-day interval of 100 µL of the fecal supernatants, 30 mg were administered by oral gavage during 15 days after *M. tuberculosis* infection.

### 2.15. Gut Microbiota Profiling

Fresh fecal pellets were collected and stored at −20 °C before extraction of total DNA. The 16S rRNA gene comprising V3–V4 regions was amplified using common primer pairs and the Illumina MiSeq platform was used to sequence DNA products of PCR amplification. The raw sequences were first quality-controlled using Quantitative Insights Into Microbial Ecology (QIIME version 2) [26] with default parameters, then demultiplexed and clustered into species-level (97% similarity) operational taxonomic units (OTU). OTU generation is based on Greengenes database (greengenes.secondgenome.com) and the reference-based method with SortMeRNA [27]. Strain composition analysis, alpha diversity analysis and beta diversity analysis were performed using QIIME.

### 2.16. Statistical Analysis

GraphPad Prism 6 (GraphPad Software, Inc., San Diego, CA, USA) was used for data analysis and preparation of all graphs. The data normality was evaluated by the Shapiro–Wilk normality test. An unpaired Student’s *t*-test was used to detect significant differences between two groups for parametric data, and a Mann–Whitney U-test for non-parametric data. Data from experiments with three or more groups were analyzed using the one-way analysis of variance test (ANOVA) corrected for multiple comparisons with Tukey’s post-test for parametric data and the Kruskal–Wallis test with Dunn’s multiple comparisons test for non-parametric data. Correlation analyses were performed using the Pearson’s correlation coefficient. The Chi-square test for trend test was used for categorical variables in the histological score. Microbial diversity indices were prepared with Past3 software. Permutational multivariate ANOVA (PERMANOVA) was used to compare microbial composition. Data were shown as the mean ± standard deviation (SD). Results were considered statistically significant when *p* < 0.05.

## 3. Results

### 3.1. Obesity Increases Inflammation and Bacterial Loads in the Lungs of M. tuberculosis-Infected Mice

**Figure 1:**
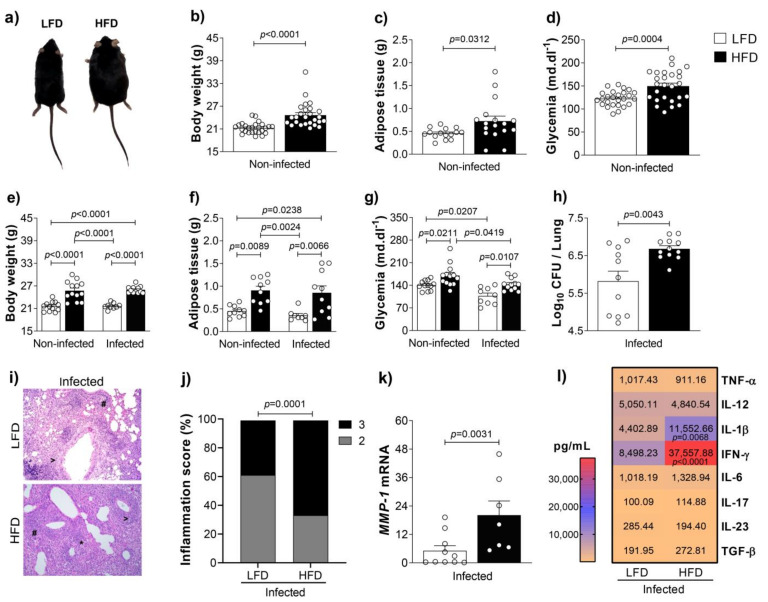
Obesity increases tuberculosis susceptibility. (**a**) C57BL/6 mice were fed with the LFD or HFD for 8 weeks and infected with *M. tuberculosis* by intra-tracheal (it) route. At 4 weeks post-infection (12 weeks of feeding), lungs were evaluated. (**b**) Body weight, (**c**) adipose tissue weight and (**d**) glycemia before infection (*n* = 15–26/group). (**e**) Body weight, (**f**) adipose tissue weight, (**g**) glycemia and (**h**) colony-forming unit (CFU) number in the lungs of lean and obese mice after infection (*n* = 8–13/group). (**i**) Representative lung sections stained with H&E (magnification 200×) (# perivascular infiltration, > peribronchial infiltration, * foamy macrophages) and (**j**) percentage of inflammation score (2 = moderate; 3 = severe). (**k**) *MMP-1* mRNA expression and (**l**) cytokine levels in lung homogenates of lean and obese infected mice (*n* = 7–10/group). Data are representative of two independent experiments and are expressed as means ± SD. Bars show the significant difference between groups (*p* < 0.05).

IFN-γ is a key cytokine that mediates protection against TB [19,20,28]. However, clinical and experimental findings reported that CD4^+^IFN-γ^+^ cells against *M. tuberculosis* antigens are directly or indirectly implicated in TB pathogenesis [21]. To assess our hypothesis that high-fat diet-induced gut dysbiosis exacerbates IFN-γ-mediated immunopathology and progression of infection, we fed animals with a low-fat diet (LFD) or a high-fat diet (HFD) for 8 weeks. Then, animals were infected or not with *M. tuberculosis* and evaluated 4 weeks post-infection. During that time, different experimental groups remained fed with the LFD or HFD, totaling 12 weeks of diet. We showed that mice fed for 8 weeks with the HFD (before *M. tuberculosis* infection) exhibited an increase of body weight and adipose tissue weight compared to mice fed with the LFD (Figure 1a–c). The HFD also induced hyperglycemia in obese compared to lean mice (Figure 1d). Four weeks or thirty days post-infection, mice fed with the HFD still exhibited a higher body weight, adipose tissue weight and glycemia compared to those fed with the LFD (Figure 1e–g). Obesity accentuated the susceptibility to *M. tuberculosis* infection and exacerbated pulmonary inflammation (Figure 1h–j). The higher expression of *MMP-1*, which is associated with tissue damage [29], in the lungs of obese compared to lean mice, reinforces the exacerbation of pulmonary pathology in the obese group (Figure 1k). In addition, we found augmented levels of IFN-γ and IL-1β in the lungs of obese compared to lean mice (Figure 1l). 

### 3.2. Obesity Accentuates the Activation and the Influx of Adaptive Immune Leukocytes to the Lungs of M. tuberculosis-Infected Mice

**Figure 2:**
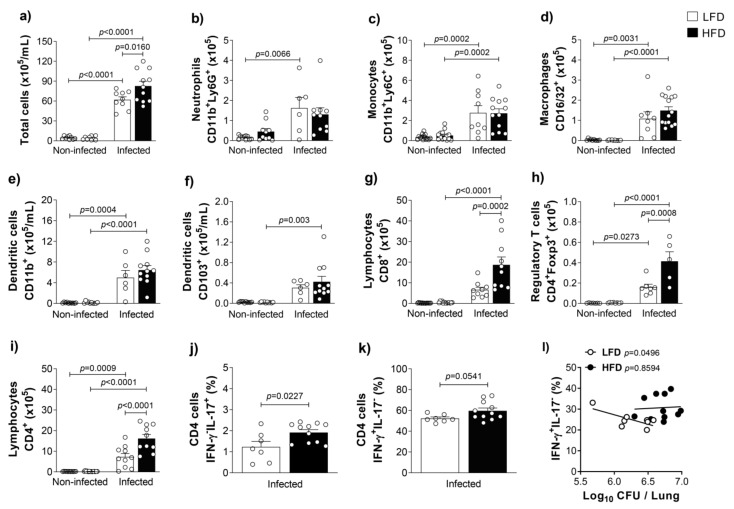
*M. tuberculosis* infection increases lung leukocyte infiltration in obese mice. C57BL/6 mice were fed with the LFD or HFD for 8 weeks and infected with *M. tuberculosis* by intra-tracheal (it) route. At 4 weeks post-infection (12 weeks of feeding), lungs were evaluated. (**a**) Total cells (×10^5^/mL) and cell number of (**b**) neutrophils, (**c**) monocytes, (**d**) macrophages, (**e**) dendritic cells CD11b^+^, (**f**) dendritic cells CD103^+^, (**g**) lymphocytes CD8^+^, (**h**) regulatory T cells Foxp3^+^, (**i**) lymphocytes CD4^+^, (**j**) IL-17-producing CD4 cells and (**k**) IFN-γ-producing CD4 cells in the lungs of lean and obese mice infected or not with *M. tuberculosis* (*n* = 6–14/group). (**l**) Correlation between CFU number and CD4^+^IFN-γ^+^IL-17^−^ cells (*n* = 8–11). Data are representative of two independent experiments and are expressed as means ± SD. Bars show the significant difference between groups (*p* < 0.05).

Since IL-1β is a pro-inflammatory cytokine and IFN-γ is the hallmark of type 1 inflammation, next, we quantified the total number and the number of different leukocyte populations in the lungs of obese or lean mice, infected or non-infected with *M. tuberculosis*. Obese and infected mice showed an increase in the total cell number in the lungs compared to lean and infected mice (Figure 2a). We detected no differences in the number of innate leukocytes evaluated in the lungs of obese or lean infected groups, which means that we found no difference in the number of neutrophils (CD11b^+^Ly6G^+^ cells, Figure 2b), monocytes (CD11b^+^Ly6C^+^ cells, Figure 2c), macrophages (F4/80^+^CD16/32^+^ cells, Figure 2d), or in the subsets of resident myeloid dendritic cells (CD11c^+^MHC-II^+^CD11b^+^CD103^−^ cells or CD11c^+^MHC-II^+^CD11b^−^CD103^+^ cells, Figure 2e,f). However, the lungs of obese and infected mice showed a significant influx of CD8^+^ cells, regulatory T cells (CD4^+^Foxp3^+^ cells) and CD4^+^ cells compared to lean and infected mice (Figure 2g–i). Furthermore, the frequencies of IL-17-producing CD4^+^ cells (CD4^+^ IFN-γ^−^IL-17^+^ cells, Figure 2j) and frequencies of IFN-γ-producing CD4^+^ cells (CD4^+^ IFN-γ^+^IL-17^−^ cells, Figure 2k) were higher in the lungs of obese animals. Interestingly, we found an inverse and significant correlation between CFU counts and frequency of CD4^+^IFN-γ^+^IL-17^−^ cells in the lungs of lean and infected mice. However, the correlation was not found in the lungs of obese and infected mice (Figure 2l). 

### 3.3. Obesity-Induced Dysbiosis Is Characterized by an Accentuated Increase of Firmicutes and Bacteroidetes in the Lungs and Firmicutes in the Stool of Infected Mice

**Figure 3:**
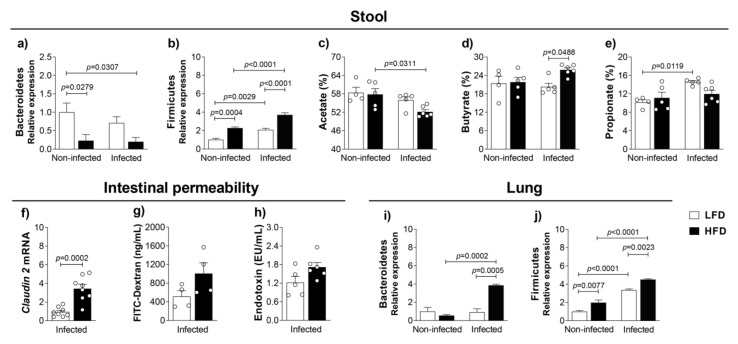
Obesity-induced dysbiosis in the lung and gut. C57BL/6 mice were fed with the LFD or HFD for 8 weeks and infected with *M. tuberculosis* by intra-tracheal (it) route. At 4 weeks post-infection (12 weeks of feeding), mice were evaluated. (**a**) Bacteroidetes and (**b**) Firmicutes relative expression from feces. Short-chain fatty acids, (**c**) acetate, (**d**) butyrate and (**e**) propionate were evaluated in stool samples (*n* = 5–6). Intestinal permeability evaluated by (**f**) relative expression of *claudin-2* mRNA in the ileum, (**g**) FITC-Dextran (ng/mL) and (**h**) endotoxin (EU/mL) in serum. Data are representative of two independent experiments for *claudin-2* and one experiment for FITC-Dextran and endotoxin (*n* = 4–8/group). (**i**) Bacteroidetes and (**j**) Firmicutes relative expression from lung samples (*n* = 7–12). Data are representative of three independent experiments for Bacteroidetes analysis and two independent experiments for Firmicutes analysis. Data are representative of two independent experiments and are expressed as means ± SD. Bars show the significant difference between groups (*p* < 0.05).

A close connection between the gut and lungs is already described [2]. Furthermore, obesity induces dysbiosis, which increases Firmicutes and decreases Bacteroidetes in the feces of obese compared to lean subjects [30], and this might increase the susceptibility to airway infections [31]. After characterizing immune response, inflammation and progression of infection in the comorbidity of obesity and TB, we evaluated the microbiota in the stool and in the lungs of lean and obese mice, infected or non-infected with *M. tuberculosis*. The analysis of microbiota in the stool confirms what has been described [32], a dysbiosis characterized by a reduction of Bacteroidetes and an increase of Firmicutes in the stool of obese and non-infected mice compared to lean and non-infected mice (Figure 3a,b). The same pattern was found, although with a significant exacerbation of Firmicutes, in the stools of obese and infected mice compared to obese and non-infected mice. Still, stools of obese and infected mice showed a significant increase of Firmicutes and reduction of Bacteroidetes compared to lean and infected mice (Figure 3a,b). We also quantified the short-chain fatty acids (SCFA), which result from the metabolism of intestinal bacteria. SCFA might contribute for the regulation of the immune response because they may collaborate for the differentiation of CD4^+^ subsets [33]. Although we found no difference in the acetate in the stools of obese and infected compared to lean and infected groups, obese and infected animals had significantly lower acetate compared to obese and non-infected animals (Figure 3c). We detected an increase of butyrate in the stools of obese and infected compared to lean and infected groups (Figure 3d). Although the stools of obese and infected mice showed a decrease of propionate compared to lean and infected animals, they were not significant, and were comparable to those detected in the stools of obese and non-infected mice (Figure 3e). Furthermore, obesity-induced gut dysbiosis might increase intestinal permeability, which can be measured by increased levels of intestinal *claudin-2*, serum FITC-dextran or serum LPS. A significant increase in the *claudin-2* mRNA in the ileum (Figure 3f), an increase of FITC-dextran (Figure 3g) and an increase of endotoxin—LPS (Figure 3h), although not significant, in the serum of obese and infected compared to lean and infected mice suggests the augmentation of intestinal permeability in the comorbidity of obesity and TB. The findings related to the gut obtained from obese infected mice show an increase of Firmicutes and butyrate and suggest an increase of intestinal permeability compared to the lean infected mice. 

We also evaluated the microbiota in the lungs. Our findings in Figure 3i,j show a significant increase of Bacteroidetes and Firmicutes relative expression in the lungs of obese and infected mice compared to lean and infected mice. These results demonstrated that *M. tuberculosis* infection also affects the microbiota and induces lung dysbiosis. We also detected a significant increase of Firmicutes, but not of Bacteroidetes, in the lungs of lean and infected mice compared to lean and non-infected mice.

### 3.4. Fecal Transfer Increases Bacterial Load and Restores IFN-γ Levels in the Lungs of Obese and Infected Mice Previously Treated With Antibiotics

**Figure 4:**
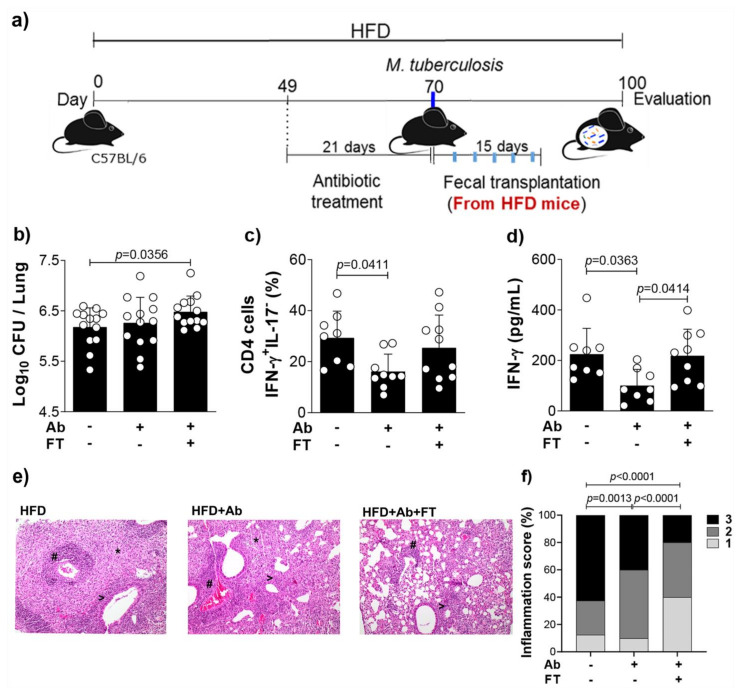
Microbiota depletion of obese infected mice reduces lung IFN-γ-producing CD4^+^ cells and IFN-γ levels. (**a**) Experimental protocol for microbiota depletion and fecal transplantation. Mice fed with HFD were treated or not with an antibiotic cocktail (Ab) for 21 days, infected with *M. tuberculosis* and transplanted (FT) or not with feces (30 mg per animal) from obese non-infected mice (5 doses, 3-day interval for 15 days). (**b**) Colony-forming unit (CFU) number, (**c**) frequency of lung CD4^+^IFN-γ^+^IL-17^−^ cells and (**d**) IFN-γ levels in lung homogenates (*n* = 8–10). (**e**) Representative lung sections stained with H&E (magnification 200×) (# perivascular infiltration, > peribronchial infiltration, * foamy macrophages) and (**f**) percentage of inflammation score (1 = mild; 2 = moderate; 3 = severe). Data are representative of two independent experiments and are expressed as means ± SD. Bars show the significant difference between groups (*p* < 0.05).

In an attempt to investigate the connection between the gut and lungs and to investigate the role of obesity-induced dysbiosis in *M. tuberculosis* infection, we depleted the gut microbiota, followed by fecal transplantation. We used 3 groups of animals: 1—HFD-fed mice infected with *M. tuberculosis* (HFD group), 2—HFD-fed mice, infected and treated with the antibiotic cocktail by gavage to deplete gut microbiota (HFD + Ab group) and 3—HFD-fed mice, infected, treated with antibiotics and transplanted with feces obtained from non-infected mice fed with HFD (HFD + Ab + FT) to restore the original experimental condition as the HFD group, as depicted in Figure 4a. The rationale for this experimental approach was to assess the hypothesis based on the reduction of both susceptibility of infection and IFN-γ-mediated immunopathology with antibiotic-depleted microbiota. In addition, fecal transfer from obese mice to obese mice previously treated with antibiotics would restore the increase of lung bacterial load and IFN-γ levels. 

Our results show that the antibiotic treatment did not affect the bacterial load as we expected (Figure 4b). However, fecal transplantation to mice previously treated with antibiotics significantly increased lung CFU counts (Figure 4b). Antibiotic treatment significantly reduced both frequency of CD4^+^IFN-γ^+^ IL-17^−^ cells and IFN-γ levels, and pulmonary inflammation (Figure 4c–f), while fecal transplantation recovered the IFN-γ levels (Figure 4d), with no effect on the pulmonary inflammation (Figure 4e,f). We also treated lean or obese mice with antibiotics and performed fecal transplantation from lean mice (Supplementary Figure S1a). Fecal transfer of lean mice to lean mice previously treated with antibiotics (LFD + Ab + FT group) significantly reduced lung bacterial load compared to lean mice (LFD group), with no effect on the frequency of CD4^+^IFN-γ^+^ IL-17^−^ cells (Supplementary Figure S1b,c). Fecal transfer of lean mice to obese mice (HFD + Ab + FT) reduced, although not significantly, the bacterial load in the lungs compared to obese mice (HFD group), and significantly reduced the frequency of CD4^+^IFN-γ^+^ IL-17^−^ cells (Supplementary Figure S1d,e). Confirming the result of the Supplementary Figure S1e, we found a decreased pulmonary inflammation in obese and infected mice that were treated with antibiotics and transplanted with feces from lean mice (Supplementary Figure S1f,h). These findings confirm that the dysbiosis as a consequence of obesity augments IFN-γ and exacerbates the pulmonary inflammation in *M. tuberculosis* infection.

### 3.5. Fecal Transplantation from Obese Mice Restores Obesity-Induced Dysbiosis 

Since lean and obese infected groups had a significant modification in the microbiota (Bacteroidetes/Firmicutes), next, we performed a deep evaluation of microbiota. 

**Figure 5:**
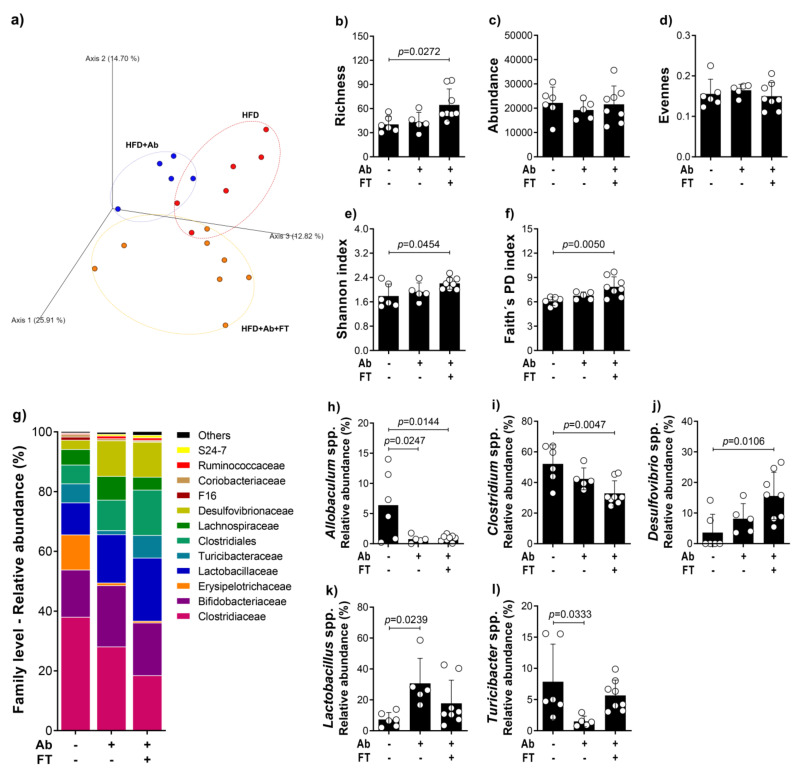
Fecal transplantation from obese mice restores obesity-induced dysbiosis. (**a**) Principal coordinate analysis (PCoA) plots based on unweighted (qualitative) phylogenetic UniFrac distance matrices, (**b**) richness, (**c**) absolute abundance, (**d**) evenness, (**e**) Shannon index, (**f**) Faith’s phylogenetic diversity index, (**g**) relative abundance at family levels and relative abundance of genera (**h**) *Allobaculum*, (**i**) *Clostridium*, (**j**) *Desulfovibrio*, (**k**) *Lactobacillus* and (**l**) *Turicibacter* of fecal bacterial OTUs. Data are representative of two independent experiments and are expressed as means ± SD. Bars show the significant difference between groups (*p* < 0.05).

The microbial community was assessed using the fecal 16S rRNA gene amplicon sequence and the identified operational taxonomic unit (OTU) was used to assess the diversity metrics. Beta diversity, represented by the principal coordinates analysis (PCoA) of the fecal bacterial community, clearly showed a difference in the microbial community between the HFD + Ab + FT in comparison to HFD and HFD + Ab groups (Figure 5a). Richness, which corresponds to the total number of OTUs, was significantly higher after fecal transplantation in comparison to obese mice that had not received the fecal transplant (Figure 5b). Depletion of the gut microbiota and fecal transplantation had no effect on the relative abundance and evenness among groups (Figure 5c,d). Shannon diversity, the most commonly reported diversity metric [34], that considers richness, abundance and evenness, was analyzed, and as expected, showed patterns similar to those seen with richness (Figure 5e). In addition, alpha diversity represented by Faith’s phylogenetic diversity was also significantly higher in the HFD + Ab + FT group in comparison to the HFD group (Figure 5f). 

To understand how the microbial community composition changed with fecal transplantation, we determined the bacterial microorganisms present at different taxonomic levels and their relative abundances. The twelve most abundant taxonomic assignments, at the family level (Figure 5g), illustrated fluctuations in the relative abundance of Clostridiaceae, Bifidobacteriaceae, Erysipelotrichaceae, Lactobacillaceae, Turicibacteraceae, Clostridiales, Lachnospiraceae, Desulfovibrionaceae, F16, Coriobacteriaceae, Ruminococcaceae and S24-7. While observable fluctuations in relative abundance levels are seen in these families, results from relative abundance at the genus level inside these families were significantly different among the HFD groups. The depletion of gut microbiota by antibiotic treatment resulted in a permanent reduction of *Allobaculum* spp. (Erysipelotrichaceae) (Figure 5h). The fecal transplantation deepens the reduction of *Clostridium* spp. (Clostridiaceae, Clostridiales) and the increase of *Desulfovribrio* spp. (Desulfovibrionaceae) as a consequence of antibiotic treatment (Figure 5i,j). Interestingly, whereas the depletion of gut microbiota significantly increased *Lactobacillus* spp. (Lactobacillaceae) and decreased *Turicibacter* spp. (Turicibacteraceae), the fecal transplantations restored the obesity-induced dysbiosis (Figure 5k,l). 

## 4. Discussion

The gut microbiota plays a protective role against TB [13]. However, the gut dysbiosis as induced by obesity might negatively impact the outcome of *M. tuberculosis* infection and the pulmonary immunopathology. In the present study, the obesity-induced gut dysbiosis increased the immunopathology, IFN-γ levels and frequency of CD4^+^IFN-γ^+^IL-17^−^ cells. Although the obesity augmented the susceptibility to *M. tuberculosis* infection, the increase in the lung bacterial load of obese mice was not directly dependent on microbiota. Interestingly, the infection by *M. tuberculosis* per se also alters the gut microbiota [10] and highlights the importance of the connection between the gut and lungs [1,35]. In this view, we also showed a dysbiosis in the lungs of lean and obese non-infected groups, and a more accentuated dysbiosis in the lungs of lean and obese infected groups. Still, our findings suggest an increase of intestinal permeability. It is noteworthy that pathogen-associated molecular patterns (PAMP) from distal sites, even in the steady state, such as the bacterial products of microbiota or of dysbiosis, might modulate local immune responses induced by foreign antigens, such as the immune response in the lungs against mycobacteria antigens [8].

Here, we showed that obesity increased the susceptibility to *M. tuberculosis* infection, the pulmonary inflammation and the influx of adaptive immune cells in the lungs of infected mice. We also showed that the obesity and TB comorbidity had the same pattern as that for dysbiosis reported previously for the obesity condition. The fecal samples of obese humans or fecal samples from mice fed with the HFD (obese mice) showed an increase of Firmicutes and a reduction of Bacteroidetes compared to the fecal samples of lean subjects [32]. A recent study showed that an increase in anti-melanoma immunity, induced by the treatment with diosgenin, a natural steroidal saponin, was characterized by a change in the microbiota, with a reduction of Bacteroidetes, and augmented intra-tumor CD4, CD8 and IFN-γ expression [36]. Additionally, in type 2 diabetic patients, a significant correlation was observed between IFN-γ and the relative abundance of Firmicutes [37]. These studies show an association between dysbiosis and modulation of Th1 inflammation. The changes in the ratio of Firmicutes and Bacteroidetes in obese and *M. tuberculosis*-infected mice might also affect the intestinal permeability and short-chain fatty acids (SCFA) contents. The SCFA are metabolites produced by the anaerobic bacteria in the intestine after fermentation of the high-fiber diet, and significantly modulate the adaptive immune response [38,39] and interfere in *M. tuberculosis* proliferation [40]. Acetate (C2) and propionate (C3) are produced by bacteria of the phylum Bacteroidetes, and butyrate (C4) is synthesized by the phylum Firmicutes [41]. Our results also show the increase of butyrate in the feces of obese and infected mice that exhibited a predominance of Firmicutes in the feces and lungs compared to lean and infected animals.

Using a comprehensive analysis of gut microbiota, we showed that the diversity of bacterial composition was different among obese groups. As expected, obese, infected and microbiota-depleted mice that received fecal transplantation (HFD + Ab + FT) over two weeks showed a significant increase in richness and diversity index. The analysis at the family and genus levels revealed some microbial taxa that may be associated with IFN-γ modulation. The fecal transplantation from obese non-infected mice increased the relative abundance of *Desulfovibrio* spp., a Gram-negative bacterium that contains LPS. An increase of Gram-negative bacterial contents in the intestine and serum LPS levels might positively regulate Th1 inflammation [42,43], as suggested by our results showing an increase in serum endotoxin levels of obese compared to lean mice. Although levels of IFN-γ were recovered after fecal transplantation, the effect of antibiotic treatment predominated given that pulmonary parenchyma was still preserved compared to obese, non-treated mice. Therefore, our results show that obesity-induced gut dysbiosis increases pulmonary inflammation and IFN-γ levels. Fecal transplantation from lean mice protected lean but not obese animals against infection. In spite of this, fecal transplantation from lean to obese mice significantly reduced the frequency of IFN-γ-producing CD4^+^ cells and pulmonary inflammation compared to the obese, non-treated group. 

The obese and infected group treated with antibiotics exhibited a better preservation of pulmonary parenchyma compared with the obese, non-treated group. Some microbial taxa may be associated with the regulation of inflammation. Commensal species, such as *Lactobacillus plantarum*, and microbiota metabolites, including indole propionic acid, are associated with the reduction of TB progression [40]. Microbiota depletion of obese infected mice reduced lung IFN-γ-producing CD4^+^ cells and IFN-γ levels, and resulted in a significant increase in the relative abundance of *Lactobacillus* spp. The direct role of *Lactobacillus* in the regulation of inflammation was associated to the differentiation of regulatory T cells and autoimmunity suppression [44,45]. In addition, *Lactobacillus* effectively dampened IFN-γ- and IL-17-induced T cell responses [46]. The fecal transplantation of gut microbiota obtained from obese non-infected mice significantly decreased the relative abundance of *Clostridium* spp. in the microbiota of mice treated with antibiotics (HFD + Ab + FT group) compared to obese mice (HFD group). Similar to *Lactobacillus*, it was demonstrated that Clostridia strains provided a TGF-β-rich environment that helped in regulatory T cell expansion and differentiation and attenuated experimental colitis and allergic diarrhea [47]. Recently, we reported that exacerbation of CD4^+^IFN-γ^+^ cell-mediated inflammation, which aggravated the progression of chronic TB, was attributed to reduced frequency of CD4^+^Foxp3^+^ cells in CCR4-deficient mice, a receptor involved in the recruitment of regulatory T cells, compared to WT animals [48]. Our study highlighted the balance between pathogen tolerance and pulmonary tolerance, as we suggest for the comorbidity of obesity and TB. Together, these studies depict the importance of investigating the induction of Treg cells and IL-10 for the use of probiotics as a therapy adjuvant for severe TB. 

## 5. Conclusions

Both dysbiosis and meta-inflammation induced by obesity might negatively impact the outcome of *M. tuberculosis* infection and the pulmonary immunopathology, and cause severe tuberculosis. In this sense, the comorbidity of obesity and tuberculosis represent a good model to investigate the importance of GLA. In the present study, obesity increased inflammation and bacterial loads in the lungs of *M. tuberculosis*-infected mice, and obesity-induced dysbiosis was characterized by an accentuated increase of Firmicutes and Bacteroidetes in the lungs and Firmicutes in the stool of infected mice. Depletion of microbiota from obese mice did not affect the lung bacterial load, but it significantly reduced both the frequency of CD4+IFN-γ ^+^IL-17^−^ cells and IFN-γ levels and pulmonary inflammation, while fecal transplantation from obese mice to obese mice previously treated with antibiotics significantly increased lung bacterial load and recovered the IFN- γ levels, with no effect on the pulmonary inflammation. Our findings showed that obesity-induced dysbiosis exacerbated IFN-γ production and pulmonary inflammation in *M. tuberculosis* infection, and led to understanding of GLA and obesity-induced dysbiosis, which might reveal targets for the treatment of pulmonary inflammatory diseases, such as host-directed therapies for severe tuberculosis.

## Data Availability

The metagenome data presented in this study are openly available in NCBI BioProject (number PRJNA682356).

## Supplementary Materials

The following are available online at https://www.mdpi.com/article/10.3390/cells10071732/s1, Figure S1: Fecal transplantation from lean mice.
