# Supplementary material for: Obesity-Induced Dysbiosis Exacerbates IFN-γ Production and Pulmonary Inflammation in the Mycobacterium tuberculosis Infection
Source: Cells. 2021 Jul 8;10(7):1732. doi: 10.3390/cells10071732 (PMC8303177; doi:10.3390/cells10071732)
Supplement: Supplementary file 1 [file cells-10-01732-s001.zip › cells-1243225-supplementary.pdf]

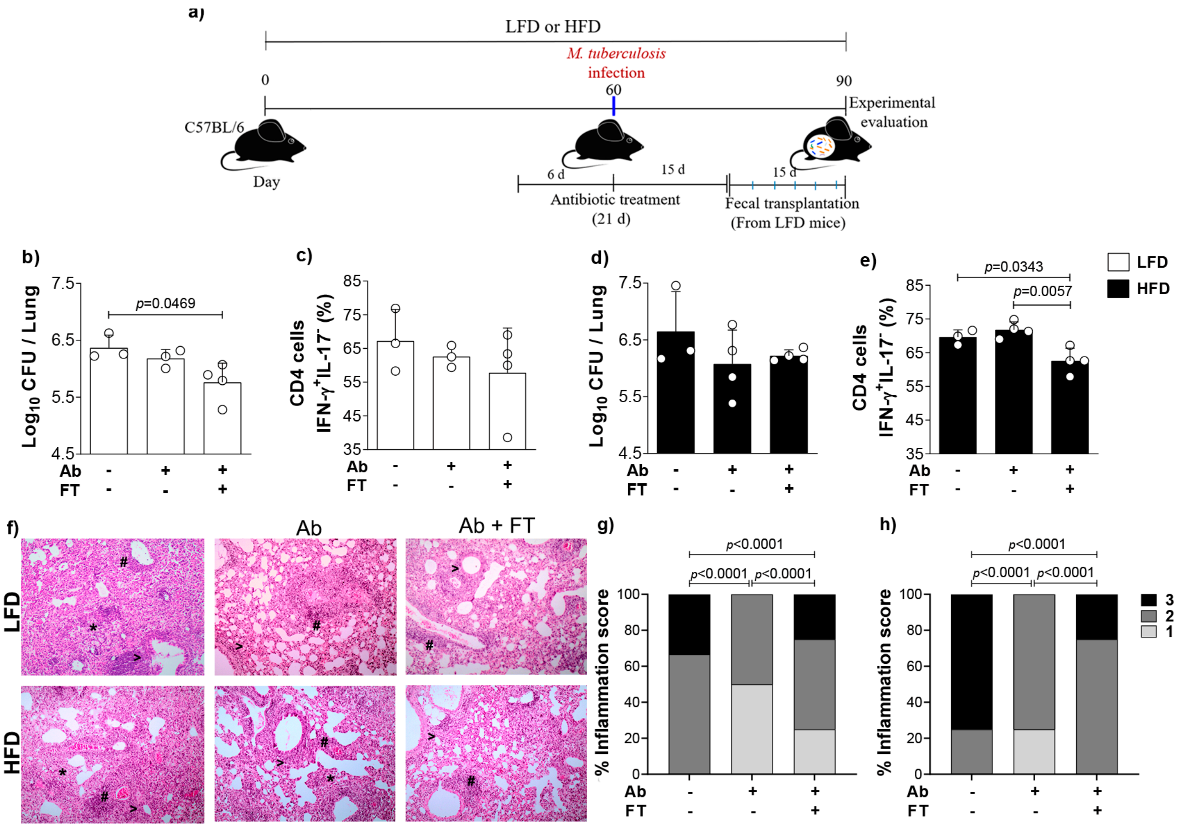


**Supplementary Figure 1. Fecal transplantation from lean mice. (a)** Experimental protocol for microbiota depletion and fecal transplantation. Mice fed with LFD or HFD were treated or not with antibiotic cocktail (Ab) for 21 days, infected with *M. tuberculosis*, and transplanted (FT) or not with feces (30 mg per animal) from lean non-infected mice (5 doses, 3-day interval for 15 days). **(b)** Colony Forming Unit (CFU) number and **(c)** frequency of lung $CD4^+IFN\text{-}\gamma^+IL\text{-}17^-$ cells in the lungs of lean infected mice. **(d)** Colony Forming Unit (CFU) number and **(e)** frequency of lung $CD4^+IFN\text{-}\gamma^+IL\text{-}17^-$ cells in the lungs of obese infected mice. **(f)** Representative lung sections stained with H&E (magnification 200 x) (# perivascular infiltration, > peribronchial infiltration, * foamy macrophages) and percentage of inflammation score (1 = mild; 2 = moderate; 3 = severe) in the lungs of **(g)** lean and **(h)** obese mice. Data are representative of one independent experiment and are expressed as means ± S.D. Bars show the significant difference between groups ($p < 0.05$).
